# Ultrafast air bonding between SiC ceramic and SnAgTi alloy under the action of ultrasounds

**DOI:** 10.1038/s41598-018-34635-w

**Published:** 2018-11-15

**Authors:** Bingzhi Wu, Xuesong Leng, Ziyang Xiu, Jiuchun Yan

**Affiliations:** State Key Laboratory of Advanced Welding and Joining, Harbin Institute of Technology, Harbin, 150001 China

## Abstract

With the aim of overcoming the limitations of traditional soldering ceramic methods for power device packaging, a simple but ultrafast bonding technology is reported. The effect and mechanism of ultrasonic action on the interfacial bonding and microstructure is investigated and thoroughly discussed. An ultrafast interfacial bond between SiC ceramics and SnAgTi active solder has been successfully achieved through a reaction at the interface at a low temperature of 250 °C in the extremely short time. High-resolution transmission electron microscopy (HRTEM) revealed that a silica layer on the surface of SiC reacted with Ti from the SnAgTi active solder to form a nanometer-thickness amorphous titania layer at the interface under the ultrasonic action, which creates an exceptional interfacial structure and facilitates bonding between the two dissimilar crystals. A discontinuous titania layer at the interface was identified within 0.1 s. With further increasing ultrasonic action time to 1 s, a continuous titania layer with a thickness of 7.6 ± 0.5 nm formed at the interface. A new interfacial reaction mechanism was revealed and it was found that ultrasound accelerated the reaction of liquid active solder/ceramic. Our finding demonstrated that ultrasound could be an effective approach for joining ceramics which is difficult to wet by a liquid metal at low temperature. The combined impact of ultrasonic cavitation and streaming dominated the mechanism and kinetics of the rapid interfacial reaction.

## Introduction

SiC ceramic, owing to their outstanding mechanical properties and superior physical properties, are promising materials for applications in microelectronic devices^1,2^. Moreover, SiC has been considered as the potential wide-band-grap semiconductors materials. In such cases, the SiC must be jointed to obtain useful and stable components considering the practical application. For example, the die attachment approach is the major interconnecting method between SiC die and the substrate, and it is an indispensable step in fabricating the SiC power electronic devices in the electronic packages^3^. As a result, the development of appropriate low-temperature joining techniques of SiC is in demand.

Soldering is a versatile technique to join a wide range of ceramics or metals at low temperatures. Electronic solders^4^, such as SnAg, BiSn, and AuSn, are widely used for the bonding of electronic components. However, these solders exhibit poor wettability with ceramics and cannot be used for the direct soldering of ceramics^5,6^. To overcome the poor bonding nature of solder on ceramic surfaces, a standard industrial process for soldering ceramics that involves a premetallization step prior to soldering has been used^7–9^. Because this technique is complex and expensive, attempts have been made to eliminate the metallization step by adopting solders containing “active” alloying elements, such as Ti, Zr or V. Ti is one of the most frequently used active constituents of Sn-based active solders^10–13^, whose role is to react with the ceramic to produce wettable products and subsequently improve interfacial bonding. However, owing to the highly covalent character of the Si-C bonds in a hexagonal crystallographic SiC lattice^14^, high temperatures (greater than 800 °C) are required to achieve a sufficient interfacial reaction between Ti and SiC^15–17^.

Based on experimental studies conducted at low temperatures, elemental Ti strongly reacts with SiO_2_. For example, Chang *et al*.^18^ have successfully performed the direct soldering of ZnS–SiO_2_ ceramic by using Sn3.5Ag4Ti at 250 °C. The same approach has been adopted by Cheng *et al*.^19^. However, although the bonding of SiO_2_ was realized at low temperature, one problem was faced, namely, a long bonding time of at least tens of minutes for a sufficient reaction between Ti and SiO_2_. Longer bonding times can lead to seriously negative effects on the reliability of the packing systems. Ultrasonic-assisted soldering has been shown to form chip interconnections at low temperatures within short bonding times^20–22^. Previous studies have demonstrated that the use of ultrasound radiation dramatically accelerated the dissolution of a metal substrate toward a liquid solder. Actually, the propagation of ultrasonic waves in a molten alloy medium results in complex ultrasonic effects and affect the physical-chemical interactions at liquid solder/solid ceramic interfaces.

In this study, we intend to achieve the bonding of SiC with the help of a reaction between Ti and SiO_2_ at a low temperature. Prior to soldering, the oxidation of a SiC ceramic was performed through exposure to air at 250 °C for 20 min, and subsequently a SiO_2_ layer was generated on the SiC surface. In such a case, the oxidized SiC was soldered using a Sn3.5Ag4Ti solder assisted by ultrasonic action. It is hypothesized that ultrasound radiation could dramatically accelerate the reaction of Ti/pre-oxidized SiC. Hence, the rapid bonding at low temperature could be realized. To the best of our knowledge, this rapid bonding has never been reported. In particular, we thoroughly discuss the bonding mechanism and how ultrasound action promoted the reaction between pre-oxidized SiC and SnAgTi solder.

## Results

### Characterization of pre-oxidized SiC

**Figure 1:**
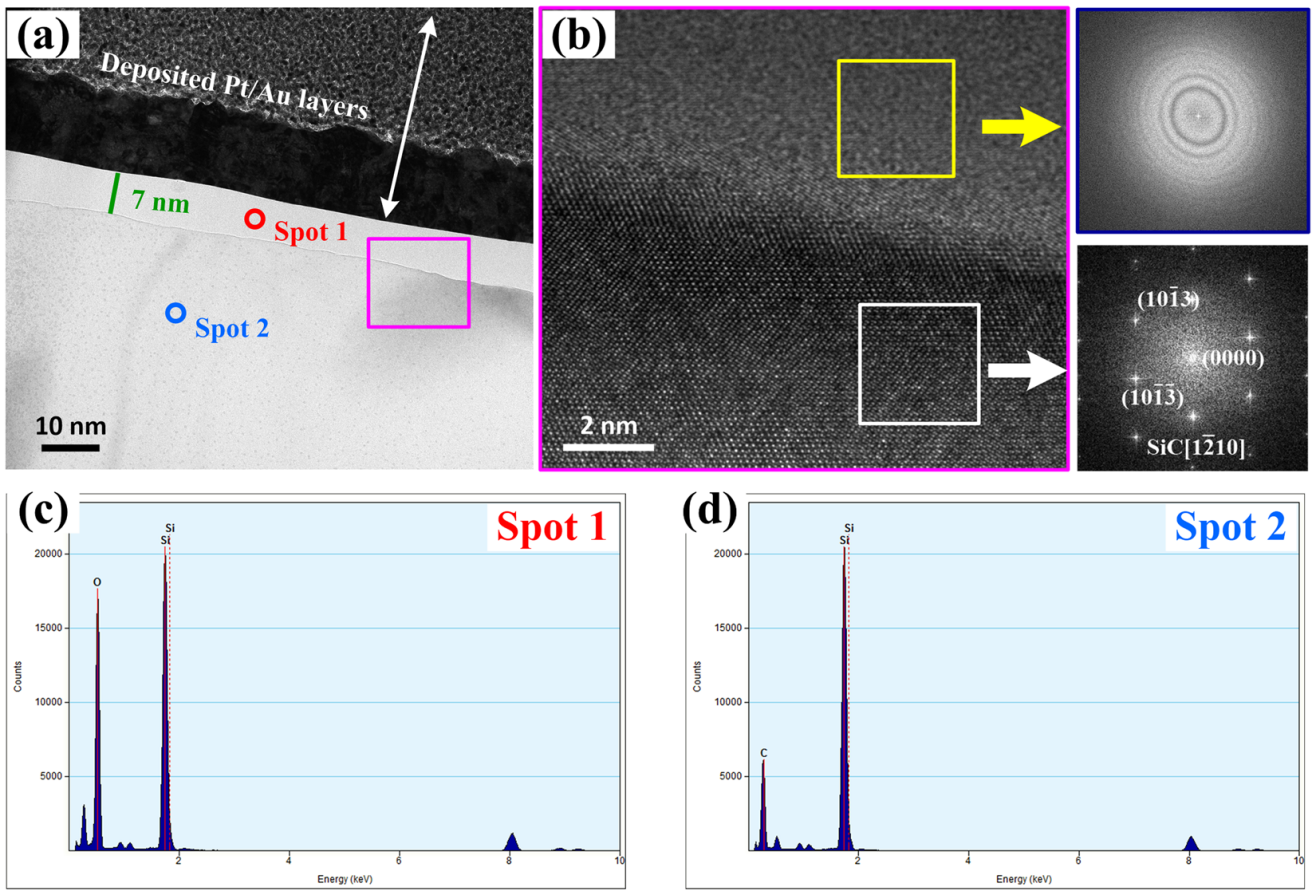
Identification of SiO_2_ on SiC surface after annealing for 20 min at 250 °C. (**a**) Bright-field cross-sectional TEM image showing a layer; (**b**) HRTEM images of the interface between SiO_2_ and SiC. The insets in (**b**) are corresponding Fourier reconstructed pattern of white rectangular region in (**b**) with SiC[1–210] and diffraction pattern obtained by fast Fourier transform (FFT) of the blue rectangular area, respectively; (**c**,**d**) nanoprobe EDX results of the same location as shown in (**a**).

SiC was firstly heat-treated at 250 °C for 20 min in order to increase its reactivity with active solder by increasing its surface energy. To characterize the fine structure of the oxide layer, transmission electron microscopy (TEM) together with HRTEM was employed. It can be seen from Fig. 1(a) that an obvious layer with a thickness of about 7 nm existed on the SiC surface. The Au and Pt layer was deposited by ion sputtering to ensure the ceramic surface could be conductive. Figure 1(b) shows the HRTEM image of interface in the area marked in Fig. 1(a) together with fast Fourier transform (FFT) image. From Fig. 1(b), it could confirm the amorphous nature of the oxide layer. In addition, nanoprobe EDX (Fig. 1(c)) have confirmed that the layer is indeed SiO_2_, which is the native oxide layer of SiC^23^, and the amorphous was at thermodynamic equilibrium due to the low annealing temperature, which was in accordance with the results obtained by Roy^24^.

### Microstructures of the joints and interfaces

**Figure 2:**
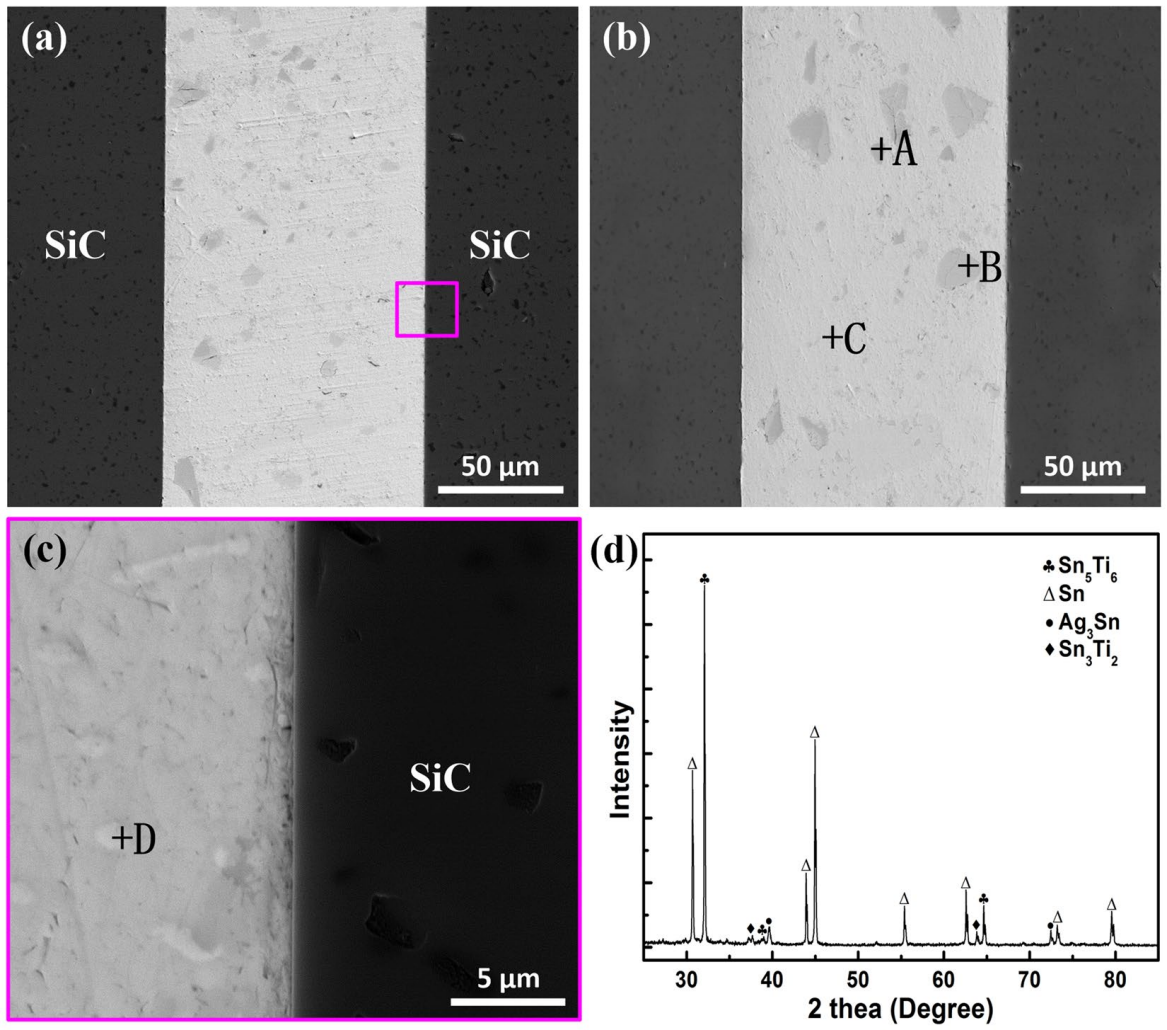
Typical SEM images of the as-formed SiC joints soldered under the ultrasonic action for (**a**) 0.1 s and (**b**) 1 s; (**c**) the corresponding magnification views as marked in (**a**); (**d**) MF-XRD patterns of the bond layer in (**a**).

Figure 2 shows the SEM images of cross-sections of SiC joints soldered with different ultrasonic action times. As shown in Fig. 2(a,b), the joints were soundly metallurgical bonded without any obvious defects, such as voids and cracks. Figure 2(c) shows the high magnification image of bond region in Fig. 2(a). The results demonstrated that the relatively good wettability of SnAgTi alloy on SiC ceramic was obtained under the ultrasonic action. The EDS results of each spots are listed in Table 1. From the EDS results and the MF-XRD patterns of the bond layer (Fig. 2(d)), it could be determined that the microstructure of the bond layer mainly consisted of four phases including a dark Sn_5_Ti_6_ phase (marked by A), a gray Sn_3_Ti_2_ phase (marked by B), a light gray Sn-rich phase (marked by C) and a white Ag_3_Sn phase (marked by D).

**Table 1:** Chemical compositions and possible phases of each spot marked in Fig. 2 (at%).

Spot	Sn	Ag	Ti	Possible phase
A	42.1	—	57.9	Sn_5_Ti_6_
B	57.7	—	42.3	Sn_3_Ti_2_
C	99.6	0.4	—	Sn-rich
D	25.8	74.2	—	Ag_3_Sn

**Figure 3:**
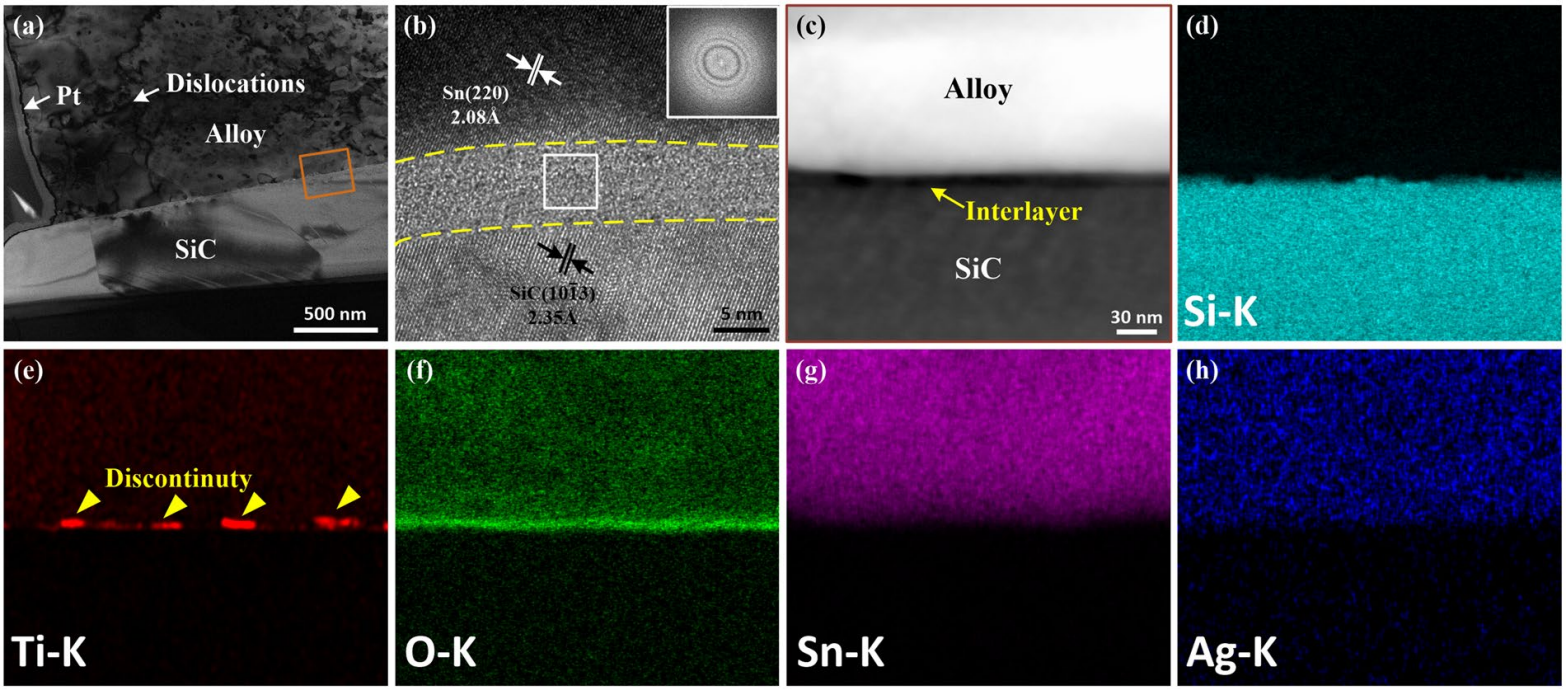
(**a**) Bright-field cross-sectional TEM image of the interface soldered with ultrasonic action for 0.1 s; (**b**) high-resolution TEM (HRTEM) image of the interface, and the inset was obtained by fast Fourier transform (FFT) of the white rectangular area; (**c**) HAADF image of the orange range in (**a**) and the corresponding STEM-EDS maps of (**d**) Si, (**e**) Ti, (**f**) O, (**g**) Sn, and (h) Ag.

Figure 3(a) shows a TEM image of the interface of a SiC joint soldered with ultrasonic action for 0.1 s. No voids are observed at the interface, which indicates that the SiC ceramics are successfully bonded using SnAgTi as the solder within an extremely short time. Several dislocations are visible within Sn-based solid solutions, indicating that plastic deformation might occur in these soft phases and is expected to absorb the detrimental stress^25^. An HRTEM investigation of the interface (Fig. 3(b)) shows a uniform thickness of the interlayer at the interface in the range of 7.2 ± 0.7 nm. Additionally, the interfacial layer did not show any lattice fringes or specific diffraction spots, indicating the amorphous nature of the interlayer. A TEM-EDS point analysis indicated the presence of Ti, O and Si in the interlayer. An HAADF image of this layer is shown in Fig. 3(c), and the corresponding STEM-EDS elemental mapping is illustrated in Fig. 3(d–h). The results indicate that the uniform distribution of elemental O and nonuniform distribution of elemental Ti and Si were found at the interface. Thus, the results suggest that the interlayer may consist of a Si-O phase and a Ti-O phase.

**Figure 4:**
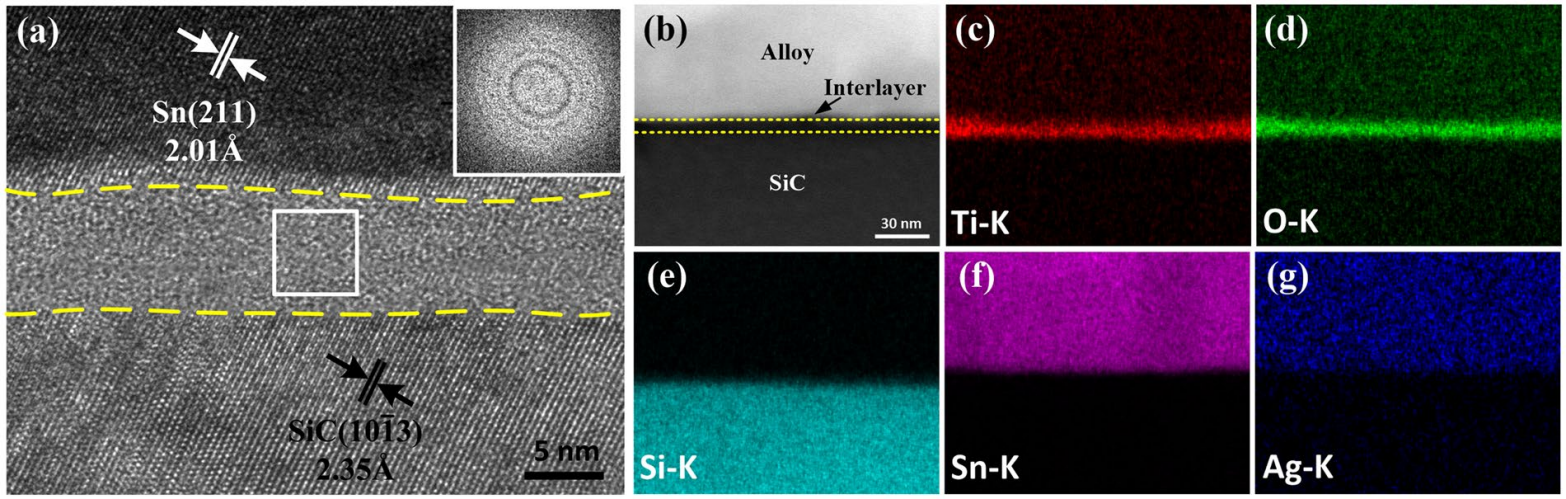
(**a**) High-resolution TEM image of the interface soldered with ultrasonic action for 1 s, and the inset was obtained by FFT of the white rectangular area; (**b**) HAADF image of the interface and the corresponding STEM-EDS maps of (**c**) Ti, (**d**) O, (**e**) Si, (**f**) Sn, and (**g**) Ag.

As shown in Fig. 4(a), when the ultrasonic action time was 1 s, an amorphous interlayer with a thickness of 7.6 ± 0.5 nm evolved at the interface. An HADDF image of this layer is shown in Fig. 4(b), alongside the corresponding STEM-EDS maps for the elements present. The EDS analysis (Fig. 4(c–g)) indicated that Ti and O were components of this layer and no other elements were present. The atomic ratio of O/Ti obtained using TEM-EDS quantitative analysis is approximately 2.0, which is consistent with the stoichiometry of TiO_2_. The results confirmed that the thin continuous layer is TiO_2_.

### Bonding strength of the SiC/SnAgTi/SiC joints

**Figure 5:**
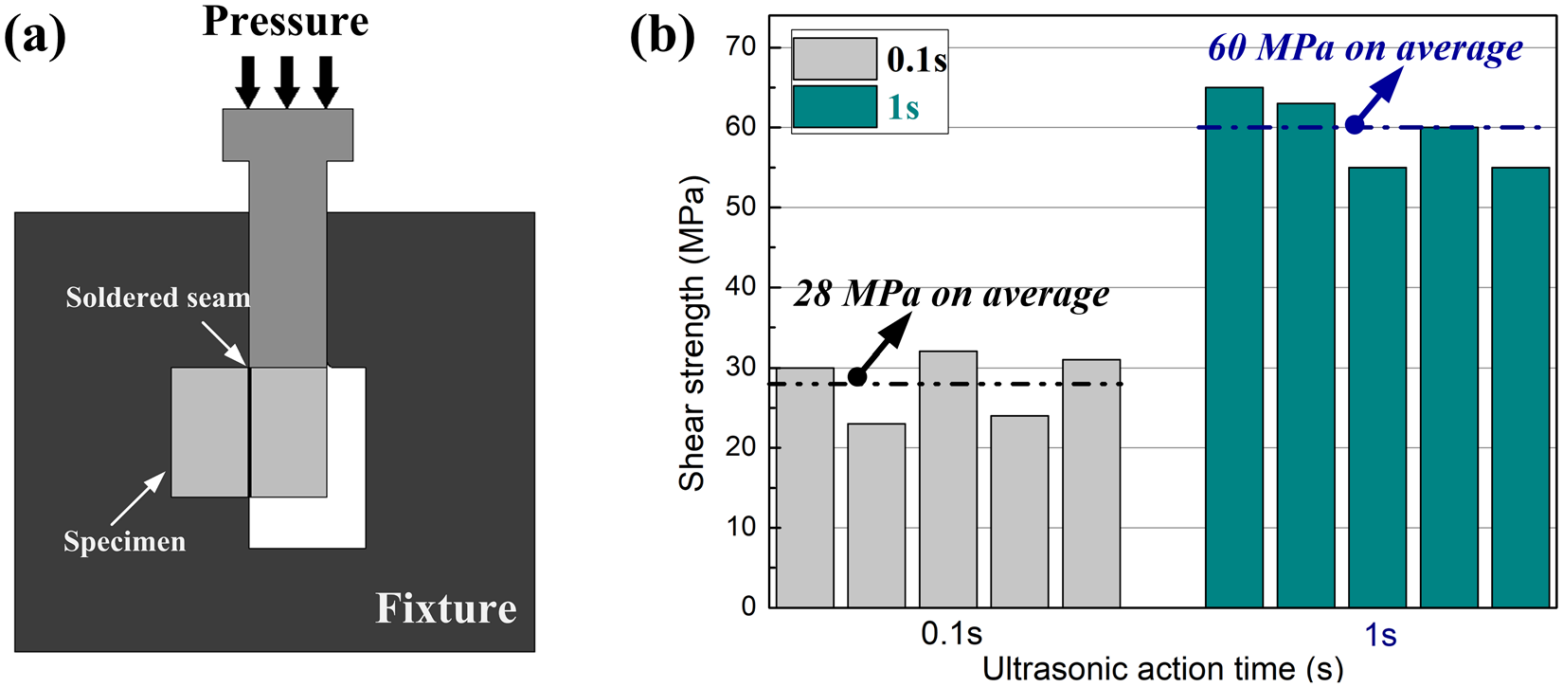
(**a**) Schematic diagram of the shear test configuration; (**b**) shear strength of SiC joints with different ultrasonic action time.

**Figure 6:**
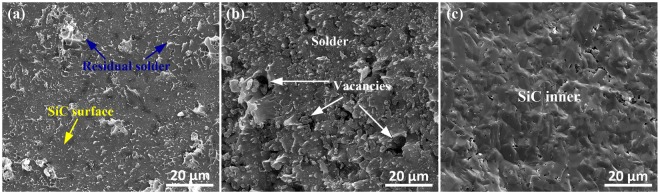
Secondary electrons SEM images of fracture surfaces of SiC joints. (**a**) At the SiC side with ultrasonic action for 0.1 s; (**b**) at the solder seam side with ultrasonic action for 0.1 s; (**c**) at the SiC side with ultrasonic action for 1 s.

As the interfacial microstructure modification, the mechanical performances must have been significant affected. A shear test was employed to characterize the bonding strength of the solder joint. The shear strength tests were measured by shear testing using a specially designed fixture as illustrated in Fig. 5(a). To ensure reliable strength results, five samples were examined at the given set of parameters to calculate the average value of the shear strength of the joints. The shear strength of the joints is shown in Fig. 5(b). From the experimental data, the results are less scatter in strength, this indicated that the bonding strength of joints is relatively stable. Figure 6 shows the SEM images of the fracture surfaces of SiC joints with different ultrasonic action times. The shear strength soldered with ultrasonic action for only 0.1 s could attain approximately 28 MPa. As shown in Fig. 6(a), protuberances attached to the fracture surface at the side were noted. EDS results indicated that the protuberances seen in Fig. 6(a) consisted of Sn-94.3 wt%, Ag-3.7 wt% and Ti-2.0 wt%. Therefore, it could be determined that the protuberances were residual solders. This observation was in agreement with the fracture surface at the solder seam side in Fig. 6(b), where some vacancies existed. It showed the bonding strength of the local interface region was enhanced. It is expected that the formed joints will satisfy applications in electronic packaging and meet the requirement for high-efficiency fabrication in electronic engineering. As the ultrasonic action increased to 1 s, a sufficient reaction between Ti and SiO_2_ occurred, thereby improving the interfacial bonding strength, and the shear strength of the joints reached about 60 MPa, which is attributed to the sufficient reaction between Ti and SiO_2_. As shown in Fig. 6(c), when the ultrasonic action time was 1 s, the typical characteristics of brittle cleavage appeared on the fracture surface, indicating the fracture occurred inside the SiC ceramic. It was also confirmed that the formation of this amorphous layer played a key role in determining the bonding properties of the joints.

## Discussion

Based on above-mentioned, after annealing the SiC in air, a SiO_2_ layer with the thickness of approximately 7 nm was generated on the surface of the SiC. When the ultrasonic action time was 0.1 s, a discontinuous Ti-O layer was formed at the interface. When the ultrasonic action time increased to 1 s, a continuous TiO_2_ layer was formed and its thickness was almost equivalent to the SiO_2_ layer. Thus, it is reasonable to infer that the formation of the interfacial TiO_2_ layer is caused by a reaction between Ti and SiO_2_. The following equation represents this redox reaction:

1 $${\rm{Ti}}({\rm{l}})+{{\rm{SiO}}}_{2}({\rm{s}})\to {{\rm{TiO}}}_{2}({\rm{s}})+{\rm{Si}}({\rm{l}})$$

**Figure 7:**
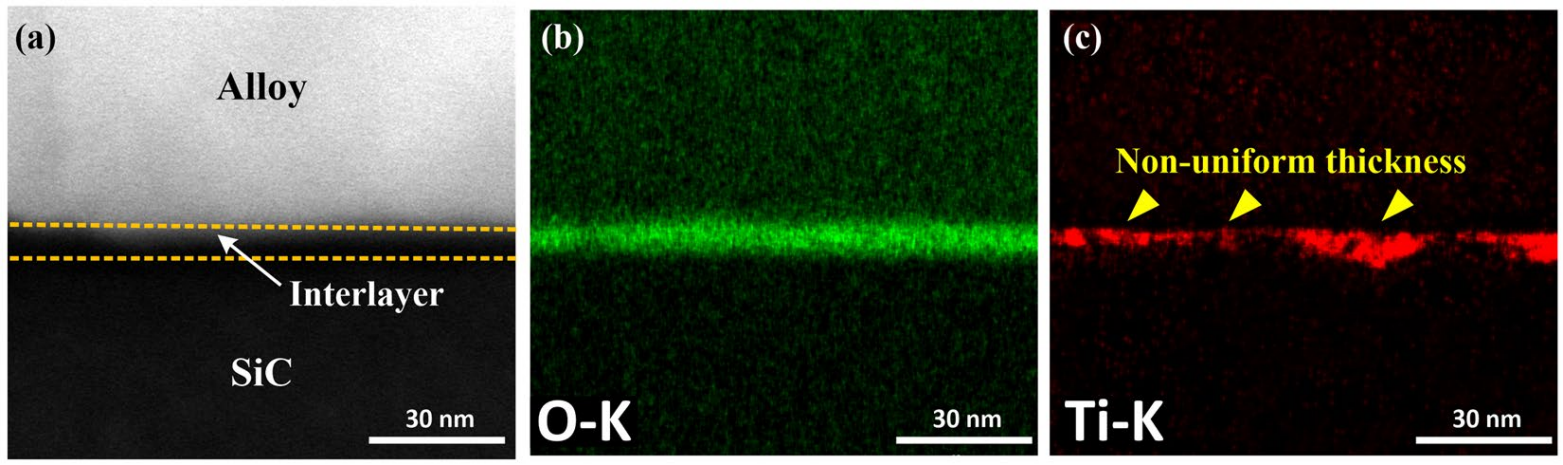
(**a**) HADDF image of the interface soldered with ultrasonic action for 0.1 s and constant temperature treatment for 5 min; (**c**,**d**) the corresponding STEM-EDS maps of O and Ti, respectively.

Considering the thermal information^26^, the variation in the Gibbs free energy of Eq. (1) is negative (−118.9 kJ mol^−1^) at 250 °C, indicating that the reduction of SiO_2_ by Ti is thermodynamically favorable. Unlike the results of other previously published papers, which reported that the reaction between Ti and SiO_2_ occurred under extremely harsh conditions, such as higher temperatures (above 900 °C)^27^ and longer holding times as long as tens of minutes^18,19^, our results indicate that a rapid reaction occurred under the following experimental conditions: a low temperature (250 °C) and an extremely short ultrasonic time (0.1 s). It is reasonable to conjecture that the reaction was facilitated by the enhanced positive effects of ultrasound radiation. To verify that ultrasonic action was critical for a rapid reaction, we examined the interfacial reaction with and without ultrasonic action. After the SiC joints were first soldered with ultrasonic action for 0.1 s, the continuous constant temperature treatment of 5 min without ultrasound at 250 °C was performed. The short ultrasonic action was to ensure that the oxide film of solder was broken by ultrasonic cavitation effects, and the SnAgTi solder was in close contact with SiC. Compared with the interface soldered with only ultrasonic action for 0.1 s (Fig. 3), one remarkable result of this process was that the variations in the morphology and thickness of the interfacial TiO_2_ were not obtained (Fig. 7). This indicates that the apparent interfacial reaction between Ti and SiO_2_ did not occur during the constant temperature treatment of 5 min. However, it was noted that a continuous and uniform thickness of TiO_2_ appears at the interface with ultrasonic action for 1 s (Fig. 4). Hence, it can be validated that ultrasonic action was indispensable for the rapid redox reaction of Ti/oxidized SiC.

During ultrasonic-assisted soldering, the propagation of ultrasonic waves in a liquid medium could generate acoustic cavitation phenomena^28,29^, which includes the formation, growth, and rapid implosive collapse of microbubbles. The heterogeneous nucleation of bubbles usually originates from the presence of submicroscopic air pockets at the solid/liquid interface on the wall of the substrate^30^. Violent bubble implosion can induce minute hot spots with a localized extreme temperature and pressure estimated to be 5000 K and 0.1 GPa, respectively^28^. Although the collapse of a single bubble only lasts for several microseconds, the bubbles confined in the thin liquid layer were imploding consecutively^31^. Thus, the bubbles imploding adjacent to the interface can generate intense heating effects on the solid surface. For a thermally activated reaction process, both the (i) diffusion of elements and (ii) reactions at the interface are driven by the localized high temperature, resulting in a rapid interfacial reaction within an extremely short time. In addition, bubble collapse leads to liquid microjets with a high velocity of few hundred meters per second and the emission of shock waves that reached a pressure of several GPa with a starting shock velocity of ~ 40000 ms^−1^ ^28^. The combined work of the microjets and shock waves results in microdamage on the SiC surface under attack, which is known as cavitation erosion^29,30^. Thus, the redox reaction was promoted by expediting the decomposition of silica. In addition, acoustic streaming refers to strong convective currents that occur when a liquid is subjected to an ultrasonic field^20–22^, which also accelerates the interfacial reaction by promoting substantial mass transport across the interface.

According to the earlier reports^27^, a reaction between Ti and SiO_2_ usually favors the formation of crystallized TiO_2_. Thus, one question naturally arises from the current study: why does amorphous TiO_2_ remain stable under our experimental conditions? The effect of homogeneous nucleation on the crystallization of amorphous TiO_2_ has been studied extensively by Ocana and co-workers^32^. They have shown that the transformation of amorphous titania to rutile occurs above 600 °C, followed by a very slow dissolution-crystallization process. Whereas under our experimental conditions such as a low soldering temperature (250 °C) and an extremely short holding time (shorter than 1 s), there is insufficient homogeneous nucleation kinetic energy for the crystallization of amorphous TiO_2_. Therefore, the uniquely amorphous TiO_2_ demonstrates thermodynamic stability at the interface. For metal-ceramic bonding, the crystal structures of the metal and ceramic are quite different; thus, it is difficult to directly form a strong crystal-to-crystal bond^33^. The amorphous structure at the heterointerface can effectively act as a “bridge” to bond the metal to the ceramic firmly. That is, the formation of amorphous TiO_2_ will be vital in strengthening the bonding of the SnAgTi alloy/SiC heterointerface for high-reliability applications.

**Figure 8:**
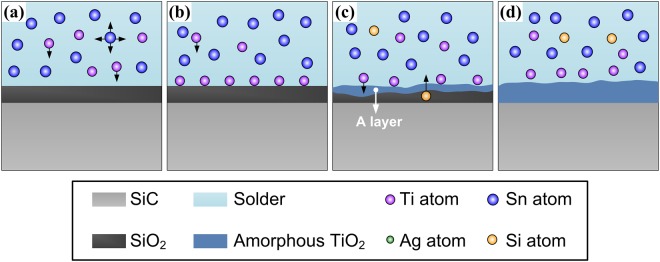
Interface evolution model for SiC/SnAgTi system. (**a**,**b**) behavior of atoms; (**c**,**d**) formation of an amorphous TiO_2_ layer.

Based on the above discussions, the Ti from the molten SnAgTi alloy has the main effect on the microstructure evolution of the joint interface. In order to analyze the microstructural evolution mechanism, a conceptual interface evolution model is established, which is schematically illustrated in Fig. 8. The whole reaction process can be divided into four stages. In the first stage, during the soldering, when the soldering temperature is up to the melting point of SnAgTi solder, the solder converts into liquid. Then, the SnAgTi liquid contacted with the SiO_2_ film on the SiC surface.

In the second stage, the Ti from the active solder diffuses towards the SiC surface and accumulated at the SiO_2_/soldering seam interface. To understand the segregation phenomena of Ti, it is necessary to analyze the adsorption force of the Ti atoms at the SiO_2_/SnAgTi interface. For oxide ceramic, due to the larger polarizability of oxygen atoms, a few atomic layer near the surface of the oxide ceramic may result in the occurrence of structure relaxation, allowing a large number of oxygen ions to occupy the outermost surface of the ceramic. Once the Ti atom arrives at the SiO_2_/SnAgTi interface, it could exchange electron with the exposed oxygen atom on the surface of SiO_2_ to form ion covalent bonds and achieve the chemical adsorption. According to the adsorption force calculation by Li^34^, the work of adhesion W can be expressed as:

2 $${\rm{W}}={{\rm{S}}}_{{\rm{w}}}\times {{\rm{n}}}_{{\rm{ws}}}$$

Where S_w_ is the slope parameter of the work of adhesion, n_ws_ and is the electron density at the boundary of the Wigner-Seitz cell of the metals. The slope parameter of SiO_2_ is about 188 mJ m^−2^ du^−1^, and the electron density of Ti is 3.51 du. The du is the density unit, and 1 du = 6 × 10^22^ electrons cm^−3^. Hence, the work of adhesion of Ti on the surface of can be calculated as follows:

3 $${\rm{W}}=188\,{\rm{mJ}}\,{{\rm{m}}}^{-2}{{\rm{du}}}^{-1}\times 3.51\,{\rm{du}}=659.88\,{\rm{mJ}}\,{{\rm{m}}}^{-2}$$

This indicates that the adsorption of SiO_2_ on Ti atoms causes the Ti atoms to constantly move toward the interface, which is the driving force leading to the Ti atoms segregating at the SiO_2_/SnAgTi interface.

In the third stage, when there is enough Ti at the interface, Ti reacts with the SiO_2_ during soldering according to reaction (1). Consequently, an amorphous titania gradually covered the SiC surface and finally formed the “A layer” as shown in Fig. 8(c). The Si atoms produced by the reaction, driven by their own concentration gradient, were liberated from the SiO_2_ layer and dissolved continuously into the molten solder at high temperature, and the dissolution could be accelerated by the ultrasonic streaming effects^20,21^. As the ultrasonic action time increased, the reaction continued and the thickness of titania adjacent to SiC ceramic grows up slowly. Since the thickness of SiO_2_ on SiC surface was tenuous, the reaction between Ti and SiO_2_ was limited, resulting in thinner titania layer at the interface finally (Fig. 8(d)). The interfacial reactions at the metal/ceramic interface play a very important role in ceramic joining. The formation of amorphous titania improved the wettability of the filler metal on the SiC ceramic and acted as the adherent layer to bond the ceramic and solder. In addition, The titania layer acts as a diffusion barrier inhibiting further reaction between Ti and SiC. Based on the Si activity diagram for Sn-Ag-Si system, once Si concentration exceeds the solid solubility limit, Si phase can be precipitated gradually from solid solution. It should be noted that, nevertheless, the existence of Si phase hardly can be observed directly in the solder seam due to its extremely low amount.

## Conclusion

In this study, an ultrafast interfacial reaction between SiC and the SnAgTi active solder was successfully achieved with the assistance of ultrasound at 250 °C. Under the action of ultrasound, a discontinuous amorphous titania layer was identified at the interface within an extremely short time (0.1 s), which was attributed to the sudden redox reaction of the active elemental Ti from SnAgTi active solder and SiO_2_ on the SiC surface. The shear strength of the joints was ~ 28 MPa which satisfies applications in electronic package. A continuous amorphous titania layer with a thickness of 7.6 ± 0.5 nm evolved at the interface under ultrasonic action for 1 s, and the shear strength of the joints reached about 60 MPa, which is attributed to the sufficient reaction between Ti and SiO_2_. The formation of an amorphous interface is crucial for promoting wetting and bonding at the SnAgTi alloy/SiC heterointerface. The propagation of ultrasonic waves in the molten solder induces ultrasonic cavitation, creating complex ultrasonic effects, which are responsible for the rapid interfacial reaction. These findings highlight the importance of the favorable sonochemical effects on the liquid solder/solid ceramic interfacial reaction, particularly with regard to the wetting and bonding of covalent SiC ceramics.

## Methods

The commercial pressureless sintered SiC ceramics (Shanghai Unite Technology Co., Ltd) were used in this study. Each of the SiC ceramics was cut into 40 mm × 10 mm × 3 mm pieces. The bonding was performed by using a Sn3.5Ag4Ti (wt%) alloy as the active solder. High purity metals of Sn (99.99%), Ag (99.999%), and Ti (99.99%) were used for alloy preparation. The solidus and liquidus temperatures of the solder are 221 and 232 °C, respectively. Prior to the assembly, all the pieces that were to be joined were ultrasonically cleaned for 15 min in acetone, and then SiC ceramics were annealed at 250 °C in air for 20 min firstly. The SiC samples were placed in a single overlap configuration with the ~20 mm overlap length. Then, the assembly of the pieces was heated in air, and the ultrasonic horn was laid on bottom of the bulk SiC. The amplitude of the ultrasonic vibration was ~3.5 μm, as measured by a laser Doppler vibrometer (Polytec OFV-505/5000, Germany). The bonding parameters of the bonding temperature, ultrasonic frequency, pressure and power were fixed as 250 °C, 20 kHz, 0.2 MPa and 500 W, respectively. The bonding time was selected as the main variable and varied in the range from 0.1 s to 1 s. The holding time at the bonding temperature was equal to the application time of the ultrasonic waves. The heating and cooling rate was approximately 30 °C/min and 15 °C/min, respectively.

The cross-sectional of soldered joints were polished for scanning electron microscope (SEM) (FEI; Quanta 200FEG) testing. The structure of the joints was investigated by micro-focused X-ray microanalysis (MF-XRD) (Bruker D8) with a beam size of 20 μm. The microstructure and energy dispersive spectrum (EDS) elemental maps of the bonded interface were characterized by transmission electron microscopy (TEM, Talos F200X) equipped with a Super-X EDS system with four silicon drift detectors (SDDs, Super-X^TM^) and a high-angle annular dark field (HAADF) microscope. Cross-sectional TEM samples were prepared by lifting out the melting zone utilizing focused ion beam (FIB) (FEI; Helios Nano-Lab 600i). Protective Pt layers were deposited over the region of interest using first the electron beam and then the Ga^+^ ion beam. Trenches were then cut through the deposits to form pre-thinned lamellae, and these were then lifted out and mounted onto copper Omni grids. The size of shear test samples of joints was 10 mm × 5 mm × 6 mm. The soldered specimens were carried out with a universal testing machine (Model 4302, Instron Ltd) at the constant speed of 0.5 mm/min at room temperature. The shear strength was calculated by the load at the fracture divided by the nominal area of the joint. The bonding strength was calculated as the peak force/fracture area. Five samples in each group were tested and the average value was used as the final results. The fracture surfaces of the joints were investigated by scanning electron microscope (SEM, FEI Quanta 200FEG).
